# Supplementary material for: Expression of Heat Shock Protein 90 Genes Induced by High Temperature Mediated Sensitivity of Aphis glycines Matsumura (Hemiptera: Aphididae) to Insecticides
Source: Insects. 2025 Jul 28;16(8):772. doi: 10.3390/insects16080772 (PMC12386264; doi:10.3390/insects16080772)
Supplement: Supplementary file 1 [file insects-16-00772-s001.zip › insects-3731721-supplementary.pdf]

Table S1 The primers used for amplification, qRT-PCR, dsRNA synthesis.

primers	gene name	5′ - 3′
PCR	*AgHsp75-F*	CCAGAACTAGCACCAGATCAGACTG
	AgHsp75-R	AAGACCAGCAAGCTCTTCTCGTTC
	AgHsp83-F	GGACTTGCCCGAAACCGATGAG
	AgHsp83-R	TACAGCCTTGTCGTTGGAATCAGC
	AgGrp94-F	GGCACAGTAACGGACAACATTAAGC
	AgGrp94-R	GGAATCGCAACAACTTGGCAAGAC
qPCR	*AgHsp75-F*	CCATTTCCAAAGAGCAACACGATG
	AgHsp75-R	AGTCTGATCTGGTGCTAGTTCTGG
	AgHsp83-F	CTGGACACGCAACCCTGATGAC
	AgHsp83-R	CGCTTGGGAATGAACAACAGTGC
	AgGrp94-F	GGCTGAATCTCCATGTGCTCTTG
	AgGrp94-R	ATCCTGACCGTAATGTTGCTGTTC
	EF1α-F	GGCTGATTGTGCTGTGCTTA
	EF1α-R	TCGCTGTATGGTGGTTCAGT
dsRNA	*AgHsp75-F*	TAATACGACTCACTATAGGGATTTTTATGA TAGTTCAACTAAAGGTG
	AgHsp75-R	TAATACGACTCACTATAGGGCGAGAAGAG CTTGCTGGTCTT
	AgHsp83-F	TAATACGACTCACTATAGGGGGACTTGCC CGAAACCGATG
	AgHsp83-R	TAATACGACTCACTATAGGGTACAGCCTTG TCGTTGGAATCA
	AgGrp94-F	TAATACGACTCACTATAGGGGGCACAGTA ACGGACAACATTA
	AgGrp94-R	TAATACGACTCACTATAGGGGGAATCGCA ACAACTTGGCAAG
	GFP-F	TAATACGACTCACTATAGGGTGAGCAAGG GCGAGGAG
	GFP-R	TAATACGACTCACTATAGGGCGGCGGTCA CGAACTCCAG

Table S2 Developmental stage-specific expression patterns of three *AgHsp90* genes

gene	*F*	df	*P*	Shapiro-Wilk *P*
AgHsp75	33.31	4	< 0.001	> 0.05
AgHsp83	8.24	4	< 0.05	> 0.05
AgGrp94	96.39	4	< 0.001	> 0.05

In Shapiro-Wilk test, the *P* > 0.05 means that the data conform to normal distribution.

Table S3 Temperature-dependent expression of *AgHsp90* genes in *Aphis glycines* after 24 h exposure to different high temperatures.

gene	stages	*F*	df	*P*	Shapiro-Wilk *P*
	N1	105.1	2	< 0.001	> 0.05
	N2	14.87	2	< 0.05	> 0.05
AgHsp75	N3	38.31	2	< 0.001	> 0.05
	N4	5.837	2	< 0.05	> 0.05
	A3	13.44	2	< 0.05	> 0.05
	N1	38.78	2	< 0.001	> 0.05
	N2	2.12	2	> 0.05	> 0.05
AgHsp83	N3	1.64	2	> 0.05	> 0.05
	N4	4.10	2	> 0.05	> 0.05
	A3	13.11	2	< 0.05	> 0.05
	N1	8.38	2	< 0.05	> 0.05
	N2	7.20	2	< 0.05	> 0.05
AgGrp94	N3	2.34	2	> 0.05	> 0.05
	N4	2.87	2	> 0.05	> 0.05
	A3	42.79	2	< 0.001	> 0.05

In Shapiro-Wilk test, the *P* > 0.05 means that the data conform to normal distribution.

Table S4 Insecticide-induced expression of *AgHsp90* genes in adult *Aphis glycines* after 24 h exposure

Insecticides	gene	t	df	P	Shapiro-Wilk P
Imidacloprid	*AgHsp75*	9.10	4	< 0.001	> 0.05
	AgHsp83	7.49	4	< 0.001	> 0.05
	AgGrp94	9.82	4	< 0.001	> 0.05
Lambda-cyhalothrin	*AgHsp75*	20.06	4	< 0.001	> 0.05
	AgHsp83	3.38	4	< 0.05	> 0.05
	AgGrp94	4.99	4	< 0.001	> 0.05

In Shapiro-Wilk test, the $P > 0.05$ means that the datas conform to normal distribution.

Table S5 Expression of *AgHsp90* genes in adult *Aphis glycines* following dsRNA feeding

gene	times	t	df	P	Shapiro-Wilk P
	24 h	23.86	4	< 0.001	> 0.05
AgHsp75	48 h	20.43	4	< 0.001	> 0.05
	72 h	3.13	4	< 0.05	> 0.05
	24 h	4.50	4	< 0.05	> 0.05
AgHsp83	48 h	6.08	4	< 0.05	> 0.05
	72 h	6.59	4	< 0.001	> 0.05
	24 h	3.11	4	< 0.05	> 0.05
AgGrp94	48 h	20.03	4	< 0.001	> 0.05
	72 h	3.18	4	< 0.05	> 0.05

In Shapiro-Wilk test, the $P > 0.05$ means that the datas conform to normal distribution.

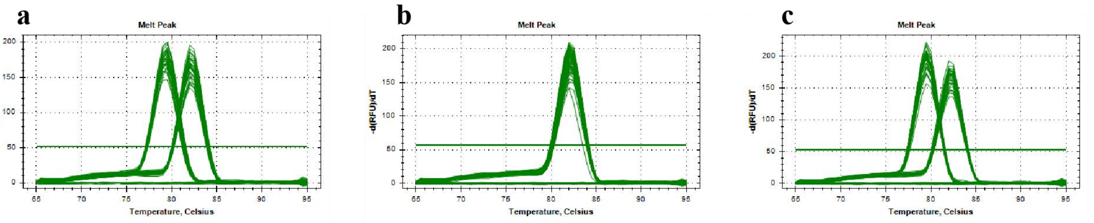

Figure S1 The melt curve of *AgHsp90s* products. *AgHsp75* (a), *AgHsp83* (b), *AgGrp94* (c).
